# Lung infections in cardiac transplant recipients

**DOI:** 10.1186/1471-2334-13-S1-O26

**Published:** 2013-12-16

**Authors:** Brînduşa Ţilea, Carmen Lucia Chiriac, Anca Meda Georgescu, Nina Șincu, Mihaela Ispas, Ioan Ţilea

**Affiliations:** 1 Infectious Disease Clinic I, Tîrgu Mureş County Clinic Hospital, Romania; 2 University of Medicine and Pharmacy, Tîrgu Mureş, Romania; 3 Cardiology Clinic 2, Institute of Cardiovascular Diseases and Transplantation Tîrgu Mureş, Romania; 4 Cardiac Rehabilitation Clinic, Tîrgu Mureş Emergency County Clinic Hospital, Romania

## Background

Lung infections represent the most common cause of mortality and morbidity in heart transplant recipients. The most important factors which determine the prevalence and type of infection in this category of patients are those related to type, regimen and length of immunosuppressive treatment.

The objectives of this study involve the assessment of the prevalence of pulmonary infections and the impact of the antibacterial, antiviral prophylactic treatment on the onset of different lung infections, following heart transplantation.

## Methods

Over the course of 48 months, we monitored 37 consecutive patients with cardiac transplant. Immunosuppressive therapy (cyclosporine, tacrolimus and prednisone) was administered. Post-transplant pulmonary infections were monitored. The etiological diagnosis (bacterial, viral, fungal and parasitic) was determined through the following methods: bacteriology, serum serology, sputum samples, bronchial biopsy, chest X-ray or chest CT scan respectively.

## Results

The mean age was 39 (range: 12 to 59 years). There were 28 male (75.7%) and 9 female (24.3%) patients. During the first 6 months following the transplant, 12 patients developed viral pneumonia, 6 bacterial pneumonia, 3 pulmonary aspergillosis, 2 pulmonary tuberculosis. During the study, a total of 58 cases of respiratory infections were recorded, with a rate of 1.5 episodes per patient.

## Conclusion

Different Gram positive and Gram negative bacterial infections, mainly community acquired, did not result in severe complications compared to fungal and specific infections which had a lethal outcome.

